# Research on the Interlayer Bonding and Temperature Control Optimization of Asphalt Concrete Core Wall

**DOI:** 10.3390/ma18102199

**Published:** 2025-05-10

**Authors:** Ziyang Luo, Wu Yang, Deqiang Han, Deyou Pan, Lei Yu, Tingpeng Guo

**Affiliations:** 1 College of Water Resources and Civil Engineering, Xinjiang Agricultural University, Urumqi 830052, China; 17562455734@163.com (Z.L.); 13832308611@163.com (D.P.); 2 Xinjiang Hydraulic Geotechnical and Structural Engineering Research Center, Urumqi 830052, China; 3 Corps Key Laboratory of Safety and Disaster Prevention of Dam Engineering, Urumqi 830052, China; 4 Xinjiang Kurgan Water Conservancy Hub Project Construction Management Center, Kashgar 844000, China; 15292399800@163.com; 5 Xinjiang Xingnong Building Materials Testing Co., Ltd., Urumqi 830052, China; 15099597679@163.com (L.Y.); 15276002653@163.com (T.G.)

**Keywords:** asphalt concrete core wall, low-temperature construction, interlayer bond mechanism, temperature transfer characteristics, performance compensation effect

## Abstract

In this paper, the mechanism of interlayer bonding under a low-temperature environment is systematically revealed in terms of the temperature control difficulties in the continuous multilayer construction of an asphalt concrete core wall in winter. A field simulation paving test was conducted using a temperature-controllable simulated paving system, and the key laws of the temperature transfer and mechanical property evolution were discovered by precisely regulating the surface temperature of the bonded surface (the test range covered from −5 °C to 70 °C). This study shows that a bonding surface temperature of 40 °C is a critical point of engineering importance, at which the material exhibited a unique performance compensation effect. Under this temperature condition, although the mechanical index was reduced compared with the parent material, the flexural strength was reduced by 11.39%, the maximum bending strain was reduced by 9.65%, the tensile strength was reduced by 7.89%, the critical tensile strain was reduced by 16.11%, and the crack curvature coefficient was reduced by 10.06%. However, thanks to the unique structural reorganization characteristics of asphalt materials, these performance losses were effectively compensated, thus ensuring the stability of engineering applications. In particular, a fast rise–stable–slow decline evolution law of the interlayer temperature transfer was found, proving the existence of a temperature-adaptive interval of the bond surface. The research results not only enrich the theory of asphalt concrete interlayer bonding but also provide innovative technical solutions for the construction of water conservancy projects in cold regions. In particular, the fast rise–stable–slow drop evolution law of the interlayer temperature transfer was found, which proves the existence of a temperature-adaptive interval of the bond surface. The research results not only enrich the theory of asphalt concrete interlayer bonding but also provide innovative technical solutions for the construction of water conservancy projects in cold regions.

## 1. Introduction

Roller-compacted asphalt concrete core walls are now key impermeable structures in water conservancy projects. Their popularity is derived from the following three traits: high density, excellent impermeability, and outstanding deformation adaptability [1,2,3,4]. These advantages are particularly significant in regions with complex geological conditions. It should be noted that climate change impacts, especially temperature variations, may affect such structures. As highlighted by Remišová et al. [5], global warming is altering climatic parameters that are critical to the performance of infrastructure. However, since the construction of asphalt concrete core walls typically employs hot construction methods, with spreading and compaction carried out in layers without segmentation along the dam axis, the construction quality is particularly sensitive to the ambient temperature [6,7,8]. This issue is particularly prominent in cold regions. The Xinjiang region is located in the hinterland of the Asia–Europe continent in the northwest of China, and it has a typical temperate continental climate. As it is in the main path of the southward Siberian cold stream, the Xinjiang region has typical extreme climate characteristics and low winter temperatures, in which the northern border has an extreme low temperature of up to −49 °C. The low-temperature period lasts up to three months (January to March), and during this period, there is generally a daily temperature difference of more than 20 °C [9]. This special climatic condition seriously affects the efficiency and quality of the winter construction of asphalt concrete core walls and brings severe challenges to the winter construction of asphalt concrete core walls. Under these conditions, it is difficult to effectively maintain the interlayer bonding temperature requirement of no less than 70 °C, as specified in the “Specification for Construction of Hydraulic Roller-Compacted Asphalt Concrete” (DL/T 5363-2016) [10]. The bonding surfaces inevitably formed during the construction process directly affect the long-term stability and anti-seepage performance of the dam body [11,12], significantly impacting construction efficiency and project quality. Therefore, studying the influence of the interlayer bonding temperature on the bonding quality of asphalt concrete core walls is of great significance. Kumlai et al. [13] found that elevated temperatures significantly reduce the dynamic modulus of asphalt concrete, thereby shortening the service life of flexible pavements. Huang et al. [14] found that the cracking resistance of asphalt concrete can be significantly enhanced if the toughness of the asphalt mastic is improved and if the aggregate gradation is optimized at low temperatures. Ahmed et al. [15] found that the temperature significantly affects the mechanical response of asphalt concrete, with temperature changes having a significant effect on the horizontal tensile strain (a key indicator of fatigue damage) and the vertical compressive strain (a key indicator of rutting). Various researchers have demonstrated that [16,17,18,19] the temperature significantly affects the fracture properties of asphalt concrete and that low temperatures significantly increase the fracture toughness of asphalt concrete, and the proportion of the temperature-induced change in the fracture toughness is essentially constant over a range of temperature intervals, suggesting a consistency of the effect on the fracture properties. Saeidi et al. [20] found that asphalt concrete at low temperatures (−15 °C) exhibits linear elastic fracture behavior, and its fracture energy (G_f_) and critical energy release rate (K_IC_) decrease significantly with the decrease in temperature and increase in aging duration. Research by Wan et al. [21] shows that when the interlayer bonding surface temperature of asphalt concrete core walls drops to around 40 °C, the construction quality of the bonding surface can be ensured without artificial heating. Hakimzadeh et al. [22] proposed an interface bonding test method based on fracture energy theory as a means to evaluate the interlayer bonding performance. Their comparative analysis shows that the test results were consistent with the trend of direct tensile test data, verifying its reliability. Zhu et al. [23] prepared asphalt concrete specimens with different bonding temperatures in the laboratory and revealed the influence of temperature changes on the mechanical properties of the bonding surface through direct shear tests. The results indicate that when the bonding surface temperature was 30 °C, the strength did not significantly decrease, and the bonding quality could still be guaranteed. Cai [24] analyzed the changes in the mechanical properties of the interlayer bonding surface of core wall asphalt concrete under different layer temperatures using the water-spraying construction process through small-beam bending tests. It was concluded that the strength and strain at the bonding surface decreased with the reduction in the layer temperature, and this process did not affect the quality of the asphalt core wall. Han et al. [25] conducted bending tests on asphalt concrete specimens at 2 °C, analyzing the bending performance at different interlayer bonding temperatures. The study showed that if the lower bonding surface temperature was above 10 °C, no heating measures were needed, providing a theoretical basis for the construction of asphalt concrete core walls under low-temperature conditions. Wang et al. [26] conducted fixed 45° angle shear tests on specimens with different bonding temperatures and concluded that when the bonding surface temperature was above the asphalt softening point, subsequent rolling construction could be carried out without reaching 70 °C. He et al. [27] studied the construction process of core wall rolling layers and found that using high-temperature hot aggregate heating could significantly improve the temperature effect of the overwinter surface base layer. Existing studies have relied mainly on indoor tests to reveal the effect of static temperature thresholds on bond quality, but there are obvious limitations to this approach. Firstly, these tests fail to replicate the complete temperature evolution pattern (rapid rise → stabilization → slow decline) induced by dynamic heat transfer during real construction. Second, they fail to capture the compensating effects of material properties that occur under real thermal changes. Most critically, current methods ignore the phenomenon of temperature-adaptive compartmentalization, i.e., bonded surfaces autonomously regulate their temperature through intrinsic heat transfer and environmental exchange. Due to these limitations, laboratory conditions cannot adequately represent dynamic heat transfer processes in complex engineering environments. Therefore, there is an urgent need to study the evolution of the dynamic temperature field and its engineering response characteristics through field experiments and then provide more scientifically based regulation strategies for practical engineering.

Based on this, this study breaks through the traditional research paradigm and focuses on the low-temperature rolling testing of asphalt concrete in the field. By systematically monitoring the temperature change patterns and mechanical performance evolution of different temperature bonding surfaces, the aim is to construct a complete research system from theory to practice. The specific objectives include the following: (1) revealing the fast rise–stable–slow decline three-stage evolution law of the interlayer temperature transfer in asphalt concrete; (2) clarifying the self-adaptive interval of the bonding surface temperature and its correlation mechanism with performance compensation effects; and (3) establishing a temperature control optimization system based on a critical temperature of 40 °C. The research results can not only provide a scientific basis for the temperature control of the bonding surface in the construction of asphalt concrete core walls in similar projects but also effectively shorten the construction period, improve project quality and efficiency, and provide important theoretical support and practical guidance for the construction of asphalt concrete core wall dams in complex environments, with significant academic value and engineering significance.

## 2. Test Raw Materials and Mix Proportions

The optimal mix ratio for the ongoing water conservancy hub project in Akto County, Kashgar Prefecture, southwestern Xinjiang, was selected for testing, with the rock being dark gray sandy limestone, where the maximum particle size was taken as 19 mm. All technical indicators of the raw materials met the requirements of SL 501-2010 “Design Specifications for Asphalt Concrete Facing and Core Wall of Earth and Rockfill Dams” [28]. The gradation index was 0.39, the asphalt-to-aggregate ratio was 7.1%, and the filler content was 13%. The asphalt used was 90# (Grade A) road petroleum asphalt produced by PetroChina Karamay Petrochemical Co., Ltd. (Karamay, China). On-site simulation of low-temperature paving tests for asphalt concrete was conducted in the field compaction test section raw material inspection, as shown in Table 1.

## 3. Test Program

On-site asphalt concrete simulated paving tests were conducted to study the bonding effect of the core wall interlayer interface under different temperature conditions (70 °C, 60 °C, 50 °C, 40 °C, and −5 °C, where −5 °C represents the overwintering surface of the asphalt concrete core wall). The placement temperature of the asphalt mixture was controlled at 170 °C, with an initial rolling temperature at 145 °C. After the paving and rolling were completed, positioning grooves were dug on the surface of the lower layer of the asphalt concrete, and temperature sensors were embedded at a depth of approximately 20 mm to monitor the temperature changes in the lower layer in real time. Once the core wall cooled to the preset temperature, the next layer of asphalt concrete core wall was paved, and the temperature changes were recorded every 30 min to monitor the temperature rise effect of the upper layer on the lower layer. After the construction of the asphalt concrete core wall in the test area was completed and fully cooled to near-ambient-temperature conditions, core drilling samples were taken, and relevant mechanical performance tests were subsequently conducted.

### 3.1. Interface Temperature Monitoring Test

**Figure 1:**
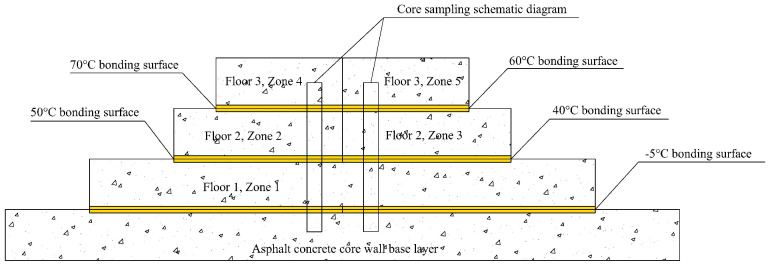
Layout of the test area.

The simulation test was divided into three layers and five zones. The first layer, 30 m in length, is designated as Zone 1, where the asphalt concrete was paved and compacted at a bonding layer temperature of −5 °C (without surface heating). The second layer, 20 m in length, is divided into Zone 2 and Zone 3. In this zone, paving and compaction were performed on the surface of the asphalt concrete after the first layer, with the surface temperature (bonding layer temperature) of the first layer controlled at 50 °C and 40 °C, respectively. The third layer, 15 m in length, is divided into Zone 4 and Zone 5. In this zone, paving and compaction were performed on the surface of the asphalt concrete after the second layer, with the surface temperature (bonding layer temperature) of the second layer controlled at 70 °C and 60 °C, respectively. Temperature sensors embedded before paving were used to accurately capture the dynamic changes in the bonding layer temperature during construction, analyzing its three-stage pattern of rapid rise, stabilization, and slow decline and verifying the existence of a temperature-adaptive range. The specific layout of the test zones is shown in Figure 1 (the yellow areas represent the bonding layer temperature monitoring zones).

### 3.2. Physical and Mechanical Property Test

**Figure 2:**
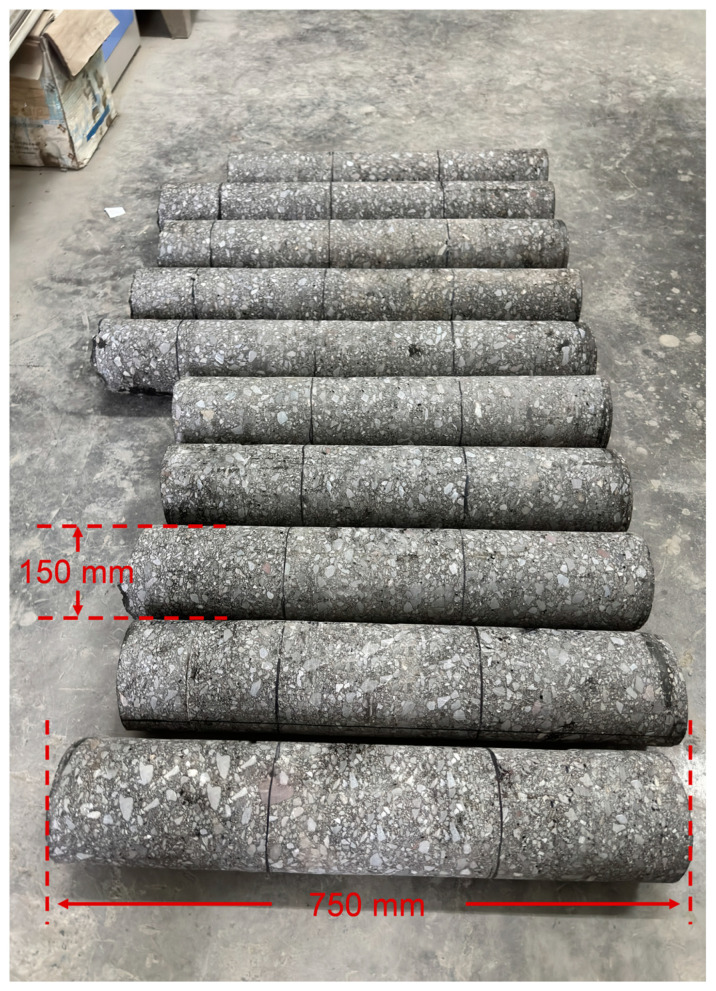
General view of core samples taken in the field.

**Figure 3:**
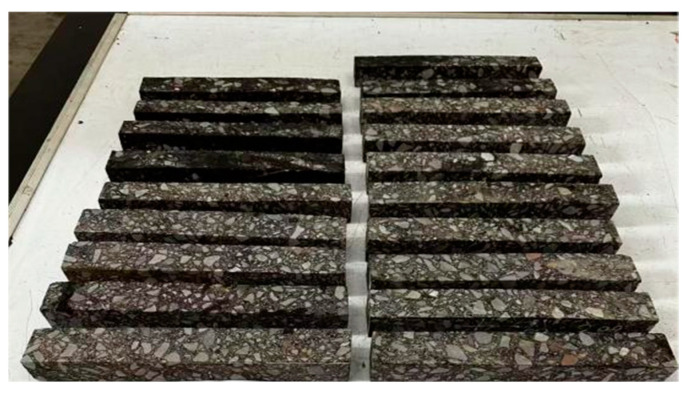
Trabecular bending specimen.

**Figure 4:**
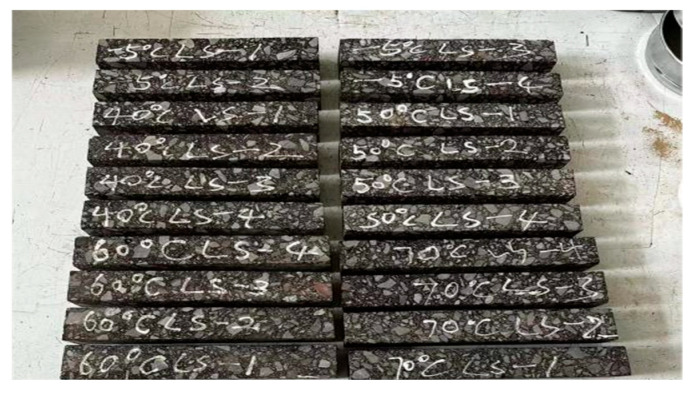
Tensile specimens for beams.

**Figure 5:**
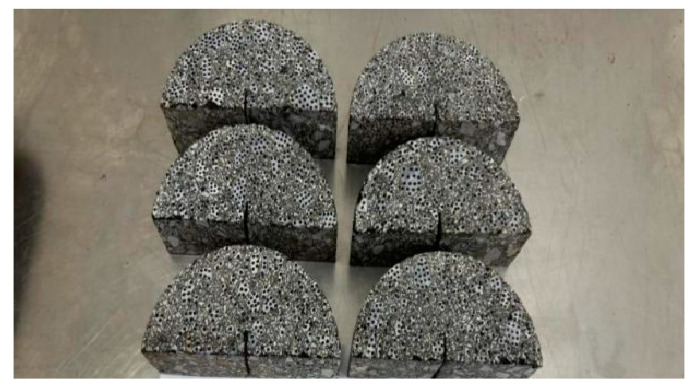
SCB specimen.

The field-obtained core samples (Figure 2) were processed into three specimen types for analysis: bending specimens (250 mm × 40 mm × 35 mm, Figure 3), tensile specimens (220 mm × 40 mm × 40 mm, Figure 4), and semi-circular bending (SCB) specimens (Ø150 mm × 25 mm thickness with 15 mm precut notches of 0.8 mm width, Figure 5). Five bonding surfaces at −5 °C, 40 °C, 50 °C, 60 °C, and 70 °C, as well as the base material, were prepared, with three specimens cut from each bonding surface, totaling 18 specimens. After specimen processing, density and porosity measurements were first conducted. Then, the specimens were accurately controlled at the required test temperature, i.e., the average annual air temperature of the project site, in accordance with the method specified in the “Test Procedure for Hydraulic Asphalt Concrete” (DL/T5362-2018) [29].

## 4. Test Results and Analysis

### 4.1. Temperature Monitoring Results and Analysis

**Figure 6:**
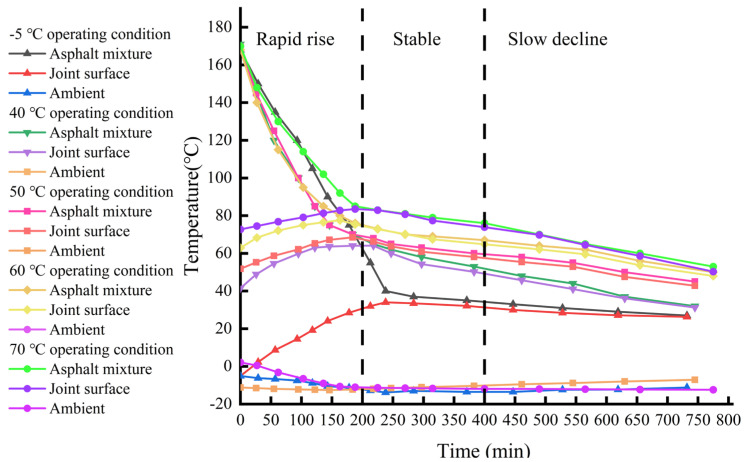
Temperature change rule of combining surface of heart wall with different temperatures.

The temperature variation patterns of the interlayer bonding surface at different temperatures are shown in Figure 6. Under the influence of heat transfer, the temperature of the lower asphalt concrete layer exhibits significant dynamic changes. According to the trend of the curves in the figure, its evolution process can be divided into three stages: rapid rise, stabilization, and slow decline. The first 200 min constitute the rapid rise stage, where the upper hot material quickly releases heat, causing the bonding surface temperature to rise sharply, with heat conduction dominating the temperature field distribution. From 200 to 400 min is the stabilization stage, where heat transfer and heat dissipation gradually balance, the temperature fluctuation range narrows, and a dynamic thermal equilibrium is formed. After 400 min, the slow decline stage begins, dominated by atmospheric temperature, during which the temperature slowly decreases and stabilizes. Our study further extends this understanding by analyzing the temperature-adaptive range and its engineering significance in more detail. In addition, our results are consistent with the observations of Zhu et al. [23], who noted that the temperature evolution mode significantly affects the mechanical properties of bonded surfaces. Internationally, Kumlai et al. [13] found similar temperature evolution patterns and emphasized the importance of temperature control during asphalt concrete construction on its performance.

When the base layer temperature was −5 °C, due to the excessively low temperature of the entire base layer, the heat transferred to the base surface after the laying and compaction of the upper hot-mix asphalt concrete was largely absorbed by the ambient air. The maximum temperature at the bonding layer was only 34 °C, significantly lower than the 70 °C required by the specifications.

Before the second layer was laid, the surface temperature of the base layer in Zone 2 was controlled at 50 °C, and that in Zone 3 was controlled at 40 °C. Since the base surface already had a certain temperature, the heat loss from the upper hot-mix asphalt concrete to the base layer was reduced. When the base temperature was ≥40 °C, the interface entered a temperature self-adaptive range, where the asphalt concrete dynamically adjusted the temperature field distribution through a heat capacity matching mechanism—balancing the heat exchange rate between the asphalt concrete’s heat capacity and the external environment. Although most of the heat was still absorbed by the ambient air, the maximum temperature at the bonding layer in Zone 2 of the second layer reached 68.5 °C, and in Zone 3, it reached 64.1 °C, meeting the 70 °C threshold requirement at the interface.

Before the third layer was laid, the surface temperature of the base layer in Zone 4 was controlled at 70 °C, and that in Zone 5 was controlled at 60 °C. The base surface temperature already met the 70 °C requirement, and with the additional heat transfer from the hot mix, the maximum temperature at the bonding layer in Zone 4 of the third layer reached 83.5 °C, and in Zone 5, it reached 77.6 °C, both exceeding the 70 °C threshold specified by the standards.

### 4.2. Changes in Density and Porosity

**Figure 7:**
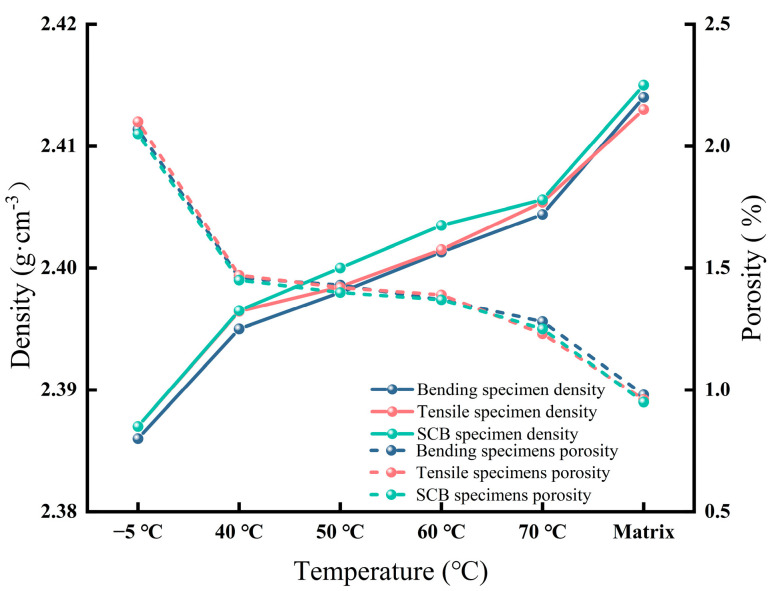
Specimen bonding surface temperature versus density and porosity curve.

The relationship between the interface temperature and the density and porosity of the asphalt concrete bending, tensile, and SCB specimens is shown in Figure 7. The test results indicate that the density of the asphalt concrete specimens decreased with the drop in interface temperature, and the porosity of the asphalt concrete did not exceed 3%, meeting the specification requirement for porosity to be less than 3%.

The test results showed that the density of the specimens showed a decreasing trend with the decrease in the interface temperature, but the porosity was always below the 3% threshold specified in the standard. The density of the specimens at 40 °C showed very little difference with the matrix material, and the porosity stabilized at below 1.5%. This phenomenon suggests that at the critical temperature of 40 °C, the asphalt concrete achieved an optimal reorganization of its internal structure through the performance compensation effect, which enhanced the densification and suppressed the growth of the porosity, thus maintaining functional stability even when the mechanical strength partially decreased. The results of this study are consistent with the finding of Cai [24], in that the density and porosity showed a similar decreasing trend as the interface temperature decreased.

### 4.3. Beam Bending Test Results and Analysis

**Figure 8:**
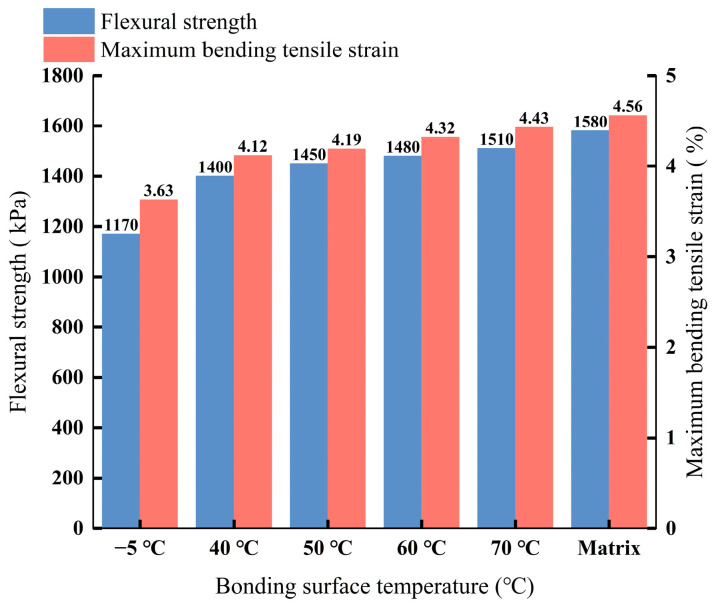
Relationship between different interface temperatures and bending strength as well as maximum tensile strain.

This test was conducted according to the predetermined experimental plan, with three specimens at each temperature. The average of the test results was taken. Bar charts showing the relationship between the bending strength, maximum tensile strain, and different interface temperatures are presented in Figure 8.

Analysis of Figure 8 reveals that the flexural strength of beam bending specimens with interfaces at different temperatures decreased as the interface temperature dropped. Flexural strength measures a material’s resistance to bending loads; higher flexural strength indicates better crack resistance under such loads. Compared to the parent material specimens, the flexural strengths of the specimens with interface temperatures of 40 °C, 50 °C, 60 °C, and 70 °C decreased by 11.39%, 8.23%, 6.33%, and 4.43%, respectively, while the specimen with an interface temperature of −5 °C showed a 25.95% reduction. Similarly, the maximum tensile strain of the beam bending specimens at different interface temperatures declined with the decrease in temperature. Tensile strain reflects the deformation resistance of asphalt concrete; higher values indicate greater deformation capacity under stress, signifying superior crack resistance. The maximum tensile strains of the specimens with interface temperatures of 40 °C, 50 °C, 60 °C, and 70 °C decreased by 9.65%, 8.11%, 5.26%, and 2.85%, respectively, compared to the parent material, while the −5 °C specimen exhibited a 20.39% reduction. Notably, although the 40 °C specimen’s flexural strength and tensile strain decreased by 11.39% and 9.65%, respectively, its density remained nearly identical to the parent material, with the porosity being stable below 2%. This suggests that at the critical 40 °C threshold, the asphalt concrete underwent internal structural reorganization, creating a performance compensation effect. Despite partial declines in mechanical indicators, it maintained functional stability through densification and interface optimization. These results are consistent with the trend of the findings of Han et al. [25]. The flexural strength and strain of the asphalt concrete were significantly increased with the increase in the bond temperature, whereas too low an interfacial temperature seriously affected its flexural properties. The performance compensation effect exhibited at 40 °C provides a scientific basis for relaxing the temperature control criteria in practical engineering.

### 4.4. Tensile Test Results and Analysis

**Figure 9:**
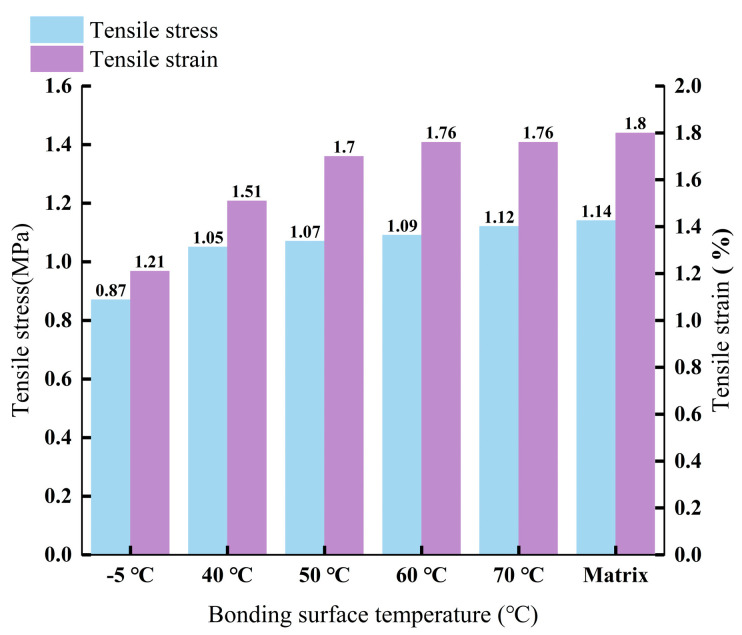
Relationship between different interface temperatures and tensile strength as well as tensile strain.

This test was conducted according to the predetermined test plan, with three specimens at each temperature. The average of the test results was taken. A bar chart showing the relationship between the tensile strength and the corresponding tensile strain at different interface temperatures is presented in Figure 9.

Analysis of Figure 9 reveals that the tensile strength of the specimens with different interface temperatures decreased as the interface temperature dropped. Compared to the base material, the specimens with interface temperatures of 40 °C, 50 °C, 60 °C, and 70 °C exhibited reductions in tensile strength by 7.89%, 6.14%, 4.39%, and 1.75%, respectively, while the specimen with an interface temperature of −5 °C showed a 23.68% decrease. Concurrently, the critical tensile strain corresponding to the maximum tensile strength also diminished with the decrease in temperature. When the interface temperature was −5 °C, the tensile strain of the specimen was relatively small, indicating that excessively low interface temperatures significantly impacted the interlayer bonding of the asphalt concrete. It can be observed that although the tensile strength of the 40 °C specimen decreased by 7.89%, its critical tensile strain retention rate remained as high as 83.89%, and the interface porosity stabilized below 2%. Internationally, Ahmed et al. [15] similarly observed this trend, stating that lower temperatures significantly reduce the tensile strength and critical tensile strain of asphalt concrete, thus affecting its interlayer bond quality. This phenomenon suggests that at the critical temperature of 40 °C, asphalt concrete maintains the continuity of interfacial bonding through performance compensation effects, effectively inhibiting interface debonding caused by stress concentration. Consequently, even with a partial reduction in mechanical strength, the ductile deformation capacity and crack resistance are preserved.

### 4.5. SCB Test Results and Analysis

To investigate the influence of the interface temperature on the bonding quality of asphalt concrete core walls, this study conducted a fracture analysis on semi-circular bending (SCB) specimens with interfaces through three-point bending tests. Hassan et al. [30] proposed the crack tortuosity coefficient, which reflects the complexity of crack propagation paths by calculating the ratio of the main crack length L*_m_* to the endpoint projection length L*_P_*. After specimen fracture, high-quality crack images were captured using an industrial camera and quantitatively processed based on the Image-Pro Plus 6.0 software: First, image calibration was performed by setting parameters such as the scale length and units to ensure measurement accuracy. Then, the “Line” tool was used to draw line segments along the crack path to measure the main crack length L*_m_* and determine the crack’s starting and ending points, measuring the endpoint projection length L*_P_*. Finally, the crack tortuosity coefficient for each group of specimens was calculated according to Equation (1).

τ = L*_m_*/L*_P_*, (1)

**Figure 10:**
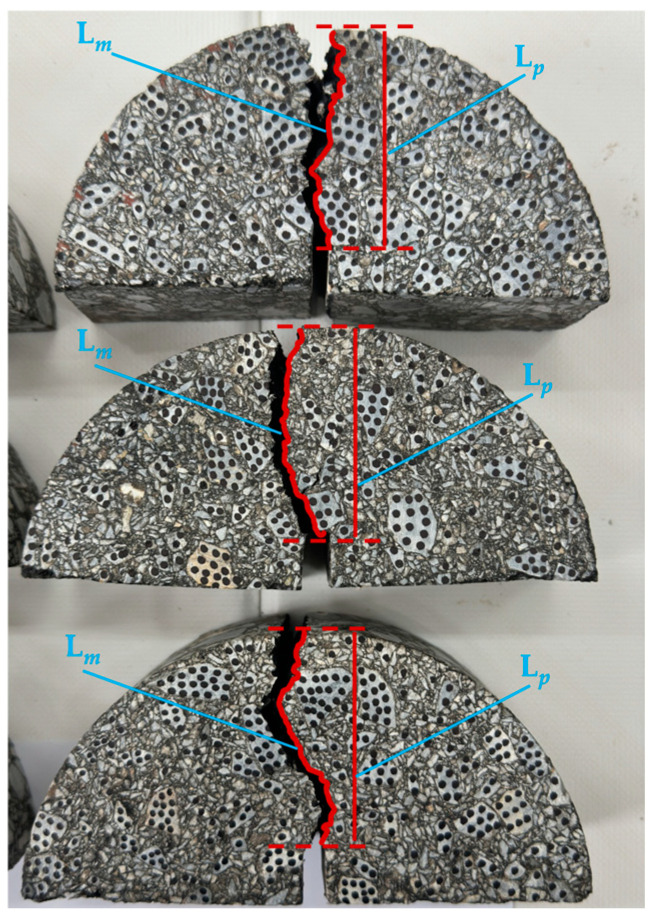
Measurement and calculation of crack curvature coefficient.

τ is the crack tortuosity coefficient (dimensionless). A higher crack tortuosity coefficient indicates better bonding quality; conversely, if the coefficient approaches 1, it indicates poorer bonding quality. By analyzing this coefficient, the bonding quality of asphalt concrete core walls under different interface temperature conditions was evaluated. Three parallel tests were conducted for each interface temperature condition to ensure data reliability. The schematic diagram of the main crack length L*_m_* and the endpoint projection length L*_P_* is shown in Figure 10.

Analysis of Table 2 reveals that the crack tortuosity coefficient decreased as the bonding surface temperature dropped. At bonding surface temperatures of −5 °C, 40 °C, 50 °C, 60 °C, and 70 °C, the median crack tortuosity coefficients were 1.375, 1.437, 1.452, 1.464, and 1.501, respectively. Compared to the base material’s value of 1.593, the reductions were 17.61%, 10.06%, 8.18%, 7.55%, and 5.03%, respectively. The range for all bonding surface temperature groups was less than 0.21, with coefficient of variation, CV, values below 9% for each group. Additionally, the maximum relative deviation, MRD, based on the median was controlled within 15%. These consistent indicators demonstrate minimal data fluctuation across the three repeated tests under the same bonding surface temperature, indicating high repeatability. The influence of temperature on the crack tortuosity coefficient was thus reliably established.

**Figure 11:**
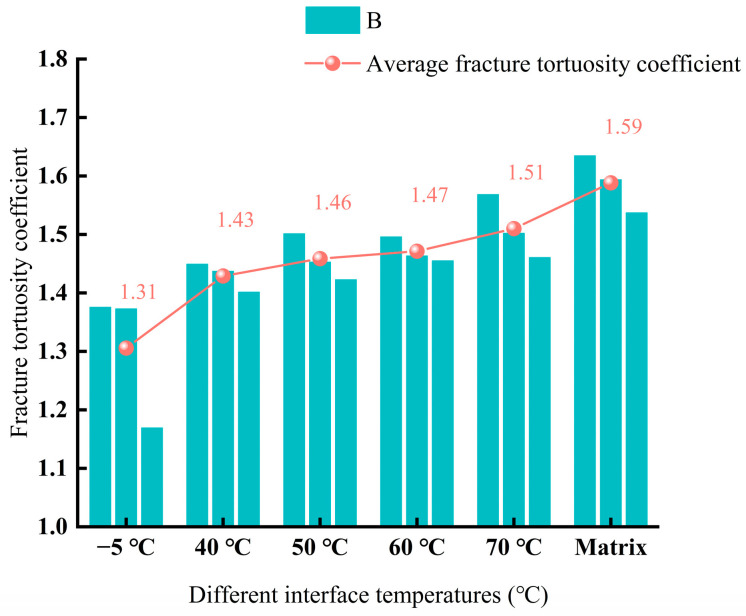
Temperature versus crack curvature coefficient for different bonding surfaces.

Analysis of Figure 11 reveals that the crack tortuosity coefficient decreased with the reduction in interface temperature. Compared to the matrix, the crack tortuosity coefficients of the specimens with interface temperatures of 40 °C, 50 °C, 60 °C, and 70 °C decreased by 10.06%, 8.18%, 7.55%, and 5.03%, respectively. The specimen with an interface temperature of −5 °C showed a 17.61% decrease in the crack tortuosity coefficient compared to the base material. This finding aligns with the research results of Kim et al. [19], indicating that the complexity of crack propagation paths in asphalt concrete is significantly affected as the interface temperature decreases. At 40 °C, the crack propagation paths exhibited multidirectional distribution, suggesting that the asphalt concrete forced the cracks to propagate tortuously by reorganizing the interface structure, thereby consuming more fracture energy. This tortuous energy dissipation mechanism is the core manifestation of the performance compensation effect. In contrast, the crack path in the −5 °C specimen was straight, forming a single dominant crack along the interface direction, which indicates that lower interface temperatures prevented the interface from compensating for performance loss through reorganization. This implies that reducing the interface temperature has a significant impact on bonding quality.

## 5. Conclusions

Based on field simulation tests and multi-dimensional characterization analysis, this study achieved the following findings in the field of low-temperature construction of asphalt concrete core walls:

(1) Through precise temperature field monitoring of asphalt concrete core layers, a three-stage evolution pattern was observed in the interlayer temperature transfer, demonstrating dynamic characteristics of rapid rise–stability–slow decline. The first 200 min represents the critical period for the rapid temperature rise. Specifically, when the temperature of the underlying core layer exceeds 40 °C, the interface exhibits a temperature self-adaptive capacity, maintaining stable thermal transfer despite ambient fluctuations. These findings provide technical support for temperature regulation during asphalt concrete core wall construction.
(2) System experiments indicate that 40 °C is the critical performance threshold temperature between layers of asphalt concrete core walls. When the temperature drops to this value, the asphalt concrete exhibits a unique performance compensation effect. Despite the decline in various mechanical indicators, the material can still maintain stable engineering performance through internal structural reorganization. This finding overturns the traditional view that when continuous paving of asphalt concrete core walls is performed, a drop in interface temperature to 40 °C will inevitably lead to a decline in core wall performance.
(3) Based on multi-dimensional experimental validation (11.39% reduction in flexural strength, 9.65% reduction in maximum bending strain, 7.89% reduction in tensile strength, 16.11% reduction in critical tensile strain, and 10.06% reduction in crack curvature coefficient), an interface temperature control evaluation system was established with 40 °C as the benchmark, reducing the traditional 70 °C temperature control standard by 30 °C. This not only significantly improves construction efficiency but also provides a new technical approach for hydraulic engineering construction in cold regions, achieving an important shift from empirical to mechanistic temperature control standards.

## Figures and Tables

**Table 1:** Raw material inspection.

Project			Normative Indicators	Actual Testing
coarse aggregate	Apparent density (g/cm^3^)		≥2.6	2.7
	Water absorption rate (%)		≤2	0.2
	Mud content (%)		≤0.5	0.2
	Durability (%)		≤12	2.2
	Crushing rate (%)		≤30	17.6
	Adhesion		≥4	Level 5
fine aggregate	Apparent density (g/cm^3^)		≥2.55	2.67
	Water absorption rate (%)		≤2	0.8
	Mud content (%)		≤2	0.3
	Durability (%)		≤15	1.7
	Water stability grade		≥6	Level 10
mineral powder	Apparent density (g/cm^3^)		≥2.5	2.6
	Hydrophilic coefficient		≤1	0.8
	Water content (%)		≤0.5	0.1
asphalt	Needle penetration 25 °C (0.1 mm)		80~100	92
	Softening point (°C)		44~52	48.2
	Elongation 15 °C (cm)		≥100	>100
	Density 15 °C (g/cm^3^)		Actual testing	0.997
	Wax content (%)		<2.2	1.8
	Solubility (%)		>99.5	99.9
	Flash point (°C)		≥245	303
	After heating the film	Mass change (%)	≤±0.8	−0.05
		Residual penetration (%)	≥57	75
		Residual elongation (cm)	≥75	>100

**Table 2:** Analysis of crack tortuosity coefficient data and its variability.

Temperature	Specimen 1	Specimen 2	Specimen 3	Median	Range	CV	MRD
Matrix	1.6343	1.5934	1.5368	1.593	0.098	3.1%	6.1%
70 °C	1.5681	1.5018	1.4603	1.502	0.108	3.5%	7.3%
60 °C	1.4956	1.4632	1.4548	1.464	0.041	1.5%	2.8%
50 °C	1.5012	1.4524	1.4223	1.452	0.079	2.9%	5.4%
40 °C	1.4488	1.4369	1.4012	1.437	0.048	1.7%	3.3%
−5 °C	1.3754	1.3725	1.1690	1.375	0.206	8.2%	15.0%

## Data Availability

The original contributions presented in the study are included in the article, further inquiries can be directed to the corresponding author.
